# Eating Alone at Each Meal and Associated Health Status among Community-Dwelling Japanese Elderly Living with Others: A Cross-Sectional Analysis of the KAGUYA Study

**DOI:** 10.3390/nu12092805

**Published:** 2020-09-13

**Authors:** Osamu Kushida, Jong-Seong Moon, Daisuke Matsumoto, Naomi Yamasaki, Katsuhiko Takatori

**Affiliations:** 1 Department of Nutrition and Life Sciences, School of Food and Nutritional Sciences, University of Shizuoka, 52-1 Yada, Suruga-ku, Shizuoka 422-8526, Japan; kushida@u-shizuoka-ken.ac.jp; 2 Department of Nutrition, Faculty of Health Science, Kio University, 4-2-2 Umaminaka, Koryo-cho, Kitakatsuragi-gun, Nara 635-0832, Japan; 3 Department of Nursing, Faculty of Health Science, Kio University, 4-2-2 Umaminaka, Koryo-cho, Kitakatsuragi-gun, Nara 635-0832, Japan; n.yamasaki@kio.ac.jp; 4 Department of Physical Therapy, Faculty of Health Science, Kio University, 4-2-2 Umaminaka, Koryo-cho, Kitakatsuragi-gun, Nara 635-0832, Japan; d.matsumoto@kio.ac.jp (D.M.); k.takatori@kio.ac.jp (K.T.)

**Keywords:** eating alone, functional capacity, community-dwelling elderly, breakfast, lunch, dinner

## Abstract

This cross-sectional study investigated the association between eating alone at each meal and health status, including functional capacity among community-dwelling Japanese elderly living with others. A self-administered questionnaire was mailed to all 8004 residents aged 65 or older, residing in the same Japanese town in March 2016. Eating alone was assessed by first asking whether participants ate three separate meals each day (i.e., breakfast, lunch, and dinner), and those who answered affirmatively were then asked how many people were usually present at each meal. Health status was assessed in terms of subjective health, medical history, care needs, body mass index, depression, and functional capacity. Data from 2809 respondents were analyzed. Those who reported not being in good subjective health and a history of hypertension were significantly more likely to eat alone at breakfast (odds ratio 1.27; 95% confidence interval 1.01–1.61, and 1.26; 1.06–1.49). Depressive symptoms and many subscales of functional capacity were also significantly associated with eating alone at breakfast, lunch, and dinner (*p* < 0.05). Many health status indicators were related to eating alone at each meal, especially breakfast.

## 1. Introduction

Population aging is a global phenomenon. In Japan, the importance of health problems in old age has drawn increased attention due to the combined factors of a declining birth rate and an aging population. The percentage of the Japanese population aged 65 years or older reached 10% in 1985, 20% in 2005, and is expected to exceed 30% in 2025 [1]. In fact, social security costs, including pension, medical, and nursing care costs, are on the rise as life expectancy increases [2]. According to the 2014 Attitude of the Elderly of Japan in the International Comparison Study, 44.9% of Japanese elderly responded that they wish to work for monetary compensation (i.e., they wish to continue working) [3]. This highlights the importance of extending healthy life expectancy for the dual purposes of a robust economy and the well-being of the elderly. These aims are in line with the Japanese term *ikigai*, which means a life worth living.

In the analyses of healthy eating behaviors, several systematic reviews have reported that the frequency of family meals is positively associated with the consumption of nutritious foods, a balanced diet, and a healthy body mass index (BMI) [4,5,6,7,8]. However, studies examining the association between family meals or eating alone and health indicators among the elderly are limited because most examinations have involved infants, children, or adolescents. A few studies involving elderly people in Japan have reported that those living alone who ate alone had poorer subjective health [9], community-dwelling elderly people who ate alone had a higher risk of depression [10,11,12], and elderly men who ate alone had a higher risk of mortality [13]. In recent years, Suthutvoravut and colleagues have suggested a relationship between frailty and living with family yet eating alone [14]. Functional capacity, such as activities of daily living and frailty, are major determinants of the elderly’s care needs [15]. Because the level of need for nursing care affects both social security costs and quality of life in the elderly, it is important to examine the association of eating alone with functional capacity and care needs.

Several studies in Israel and the UK found that eating alone is associated with poor dietary intake, such as lower energy and higher sodium intake [16,17]. Additionally, because Japanese people typically eat three meals a day, it seems likely that the situation surrounding eating alone would differ for each meal. For example, protein intake among community-dwelling elderly is higher at dinner and lower at breakfast, and it has been reported that high protein intake at lunch is associated with retention of skeletal muscle mass in men [18]. However, no studies have examined the association between eating alone and health status for each of the three meals.

If the association between eating alone and health status in the elderly is found to be different for breakfast, lunch, and dinner, then it would be possible to suggest which meal the elderly should eat with others. Thus, this study investigated the association of eating alone at each of the three meals with health status, including functional capacity, in Japanese elderly living with others.

## 2. Materials and Methods 

### 2.1. Participants

This cross-sectional study used baseline data from the Keeping Active across Generations Uniting the Youth and the Aged (KAGUYA) study. The KAGUYA study is a 5-year project to promote the integration of healthcare data and conduct “training courses for resident leaders in frailty prevention, health checks, and exercise guidance by a health support student team, and human resource development at dementia cafés” in collaboration with the town of Koryo, Nara Prefecture, and Kio University. The purpose of this project is to examine the impact of social capital on the health of residents and to use this information in constructing an effective community-based integrated care system. Although other reports have also assessed various aspects of health status, including functional capacity [19,20,21], the examination of its association with eating behavior is a novel aspect of the present study.

The baseline survey of the KAGUYA study was conducted in March 2016, using an individual self-administered questionnaire via a mail survey. The participants were all 8004 residents aged 65 years or older living in Koryo. The town, located 25 km from Osaka, has a population of more than 30,000 residents in an area of 16 km^2^, comprising rural and new residential areas. To reduce selection bias, and in consideration of the burden on the late elderly, the questionnaire stated that family members could respond on behalf of the participants. Furthermore, to improve response rates, a combination ‘thank you and reminder’ postcard was mailed to all of the participants. The questionnaire was returned by 3871 respondents (48.3% response rate).

The eligibility criteria for this study were age 65 years or older, living at home, living with others, and regularly consuming three meals daily. Accordingly, four groups of respondents were excluded from our analysis: (1) those who did not answer questions about living situations, number of people living together, whether they ate each meal, number of people eating together, and main basic characteristics (gender and age); (2) those living in nursing homes or hospitals; (3) those living alone; and (4) those who did not usually eat one or more of the three daily meals (breakfast, lunch, or dinner). Because the number of people eating together was small, we excluded those who were living alone. Additionally, we excluded those who skipped meals because the absence of eating precludes examination of the effects of eating alone or with others, which is the main target of this study.

Informed consent was obtained using the documents enclosed with the questionnaire. The protocol for the KAGUYA study was approved by the Research Ethics Committee of Kio University (approval number: H27–34).

### 2.2. Measures

The survey asked whether they usually ate three separate meals each day (breakfast, lunch, and dinner). Those who responded that they eat each meal were then asked how many people, including themselves, were present at each meal. The responses of one person were classified as *eating alone*, whereas responses of more than one person were classified as *eating together*. The KAGUYA study asked respondents about a large number of lifestyle habits that are expected to be related to health status, so we used brief binary self-reports similar to those in previous studies in Japan and the US to assess whether respondents ate alone [17,22].

Health status was examined by assessing subjective health, medical history, care needs, BMI, depression, and functional capacity.

Subjective health was classified as *in good health* for those who answered “very good,” “good,” or “not bad” to the question “How is your current health condition?” and *not in good health* for those who answered “not good” or “bad.” The same classification system was used in the Comprehensive Survey of Living Conditions of the Ministry of Health, Labour and Welfare, Japan [23].

To assess a medical history of hypertension, diabetes, cardiovascular disease, stroke, osteoporosis, rheumatoid arthritis, and dyslipidemia (high cholesterol, high triglycerides, etc.), the respondents were asked, “Have you ever been diagnosed by a doctor with any of the following diseases?”

Care needs were identified according to whether the respondents had been certified as requiring long-term care or support through the long-term care insurance system of Japan [24].

BMI was assessed to understand the low energy intake shown in previous studies [16] and was determined by asking the height (cm) and weight (kg) as integers, and dividing the weight (kg) by the square of the height (m).

Depression was assessed using the Japanese version [25] of the 5-item Geriatric Depression Scale (GDS 5) [26], a shortened version of the 15- and 30-item GDS [27,28]. The GDS 5 consists of five items, such as “Are you basically satisfied with your life?” The presence of depressive symptoms was defined as two or more negative answers to the depression screening questions. Diagnosis with GDS 5 has been proven to be significantly consistent with a clinical diagnosis of depression, with good interrater, and test-retest reliability [29].

Functional capacity was assessed using the Tokyo Metropolitan Institute of Gerontology Index of Competence (TMIG-IC) [30]. TMIG-IC is a 13-item index of competence comprising three subscales: Instrumental Self-Maintenance, such as “Can you use public transportation (bus or train) by yourself?” (5 items); Intellectual Activity, such as “Are you able to fill out forms for your pension?” (4 items); and Social Roles, such as “Do you visit the homes of friends?” (4 items). TMIG-IC measurements have high reliability according to alpha coefficients, test-retest, and the correlation between subscale and total scores, as well as high construct, discriminant, and predictive validity [31].

High levels of functional capacity are required due to changes in the living environment and improved competence of the elderly over the past quarter century, so the Japan Science and Technology Agency Index of Competence (JST-IC) was also used [32]. JST-IC is a 16-item index used to assess high-level competency, and consists of 4 subscales (each with four items): Social Engagement, such as “Do you participate in regional festivals or events?”; Technology Usage, such as “Can you use a mobile phone?”; Information Practice, such as “Are you interested in news and events from overseas?”; and Life Management, such as “Do you follow any measures to prevent yourself from becoming a victim of crime?”. JST-IC evaluations showed moderate correlations between the size of social networks and the level of subjective health well-being, and strong correlations between TMIG-IC score, physical fitness, and health literacy [33]. For both the TMIG-IC and JST-IC, each item is scored as 1 point for “Yes” and 0 points for “No.” Higher scores, therefore, indicate higher functional capabilities.

The questionnaire included socio-demographic characteristics, such as gender, age (years), education history (9 years or less, 10–12 years, 13 years or more; i.e., junior high school or less, high school, college degree or higher, respectively), self-assessed living conditions (difficult, not difficult), area of residence (former village areas, new residential areas), and working status (not working, working). The self-assessed living condition variable was classified as *difficult* for those who answered “very difficult” or “difficult” and *not difficult* for those who answered “normal,” “somewhat comfortable,” or “very comfortable” as in the Comprehensive Survey of Living Conditions of the Ministry of Health, Labour and Welfare, Japan [23]. For working status, respondents who answered “not working (including those without a regular income, pensioners, and students)” were classified as *not working*, and those who indicated “farmer,” “self-employed (self-run stores, family employment, etc.),” “working (regular),” or “working (non-regular: part-time workers, side work, etc.)” were classified as *working*.

### 2.3. Data Analysis

All data on eating alone were analyzed for breakfast, lunch, and dinner, respectively. The association between socio-demographic characteristics and eating alone was examined by binary logistic regression analysis using the presence or absence of eating alone as a dependent variable to calculate odds ratios (ORs) and 95% confidence intervals (CIs). The association between eating alone and health status was examined separately by binary logistic regression analysis when health status was binary, and by an analysis of covariance when health status was a quantitative variable. The association with health status was adjusted for possible effects of gender, age (years), education history (≤9, 10–12, ≥13), self-assessed living conditions (difficult/not difficult), area of residence (former village areas/new residential areas), and working status (not working/working) as covariates. IBM SPSS Statistics 26 (IBM Japan, Ltd., Tokyo, Japan) was used for all the statistical analyses. The level of significance was set at *p* < 0.05 (two-sided test).

## 3. Results

Of the 3871 respondents to the KAGUYA study baseline survey, the data of 2809 respondents were analyzed after applying the exclusion criteria. There were eight common reasons for exclusion: 278 respondents were residents in nursing homes or hospitals, 248 respondents were living alone, 37 did not respond to the number of people living together, 251 did not answer whether they ate each meal, 184 did not answer whether they eat each meal with others, 39 did not provide their gender or age, 20 did not usually eat breakfast, and five did not usually eat lunch. Of the 2809 respondents living with others, 796 (28.3%) ate alone at breakfast, 753 (26.8%) ate alone at lunch, and 237 (8.4%) ate alone at dinner.

Table 1 shows the association between socio-demographic characteristics and eating alone for each meal. Significant differences were reported in eating alone according to gender, age, education history, self-assessed living conditions, area of residence, and working status. In terms of gender, eating alone was more common in women than in men for breakfast (OR 1.26; 95% CI 1.07–1.48), lunch (OR 1.33; 95% CI 1.13–1.58), and dinner (OR 1.41; 95% CI 1.07–1.84). By age, eating alone was less common in those aged 70–74 and 75–79 years for breakfast (OR 0.67; 95% CI 0.54–0.83 and 0.68; 0.54–0.87) and lunch (OR 0.61; 95% CI 0.49–0.76 and 0.69; 0.55–0.88), and higher in those aged 80 years or older for dinner (OR 1.72; 95% CI 1.21–2.43) compared with those aged 65–69 years. In terms of education history, eating alone was lower in those with 10 years or more of education than in those with nine years or less for dinner (OR 0.66; 95% CI 0.48–0.90 and 0.59; 0.41–0.84). Self-assessed living conditions were lower in those with no difficulty than in those with difficulty for breakfast (OR 0.80; 95% CI 0.65–0.99). By area of residence, eating alone was lower in new residential areas than in former village areas for breakfast (OR 0.84; 95% CI 0.71–1.00) and higher for lunch (OR 1.36; 95% CI 1.15–1.61). In terms of working status, eating alone was higher in the working group than in the not working group for lunch (OR 1.24; 95% CI 1.02–1.49).

Table 2 and Table 3 show the associations between eating alone at each meal with health status. Significant differences were observed in subjective health, history of hypertension, diabetes, and dyslipidemia, depressive symptoms, and several subscales of functional capacity. For subjective health, those who reported being not in good health were more likely to eat alone at breakfast (OR 1.27; 95% CI 1.01–1.61). In terms of medical history, hypertension was higher in those eating alone than in those eating breakfast with others (OR 1.26; 95% CI 1.06–1.49), diabetes was higher in those eating lunch alone (OR 1.38; 95% CI 1.08–1.75), and dyslipidemia was lower in those eating lunch alone (OR 0.80; 95% CI 0.67–0.96). Depressive symptoms were higher in those eating alone for breakfast (OR 1.71; 95% CI 1.40–2.09), lunch (OR 1.43; 95% CI 1.17–1.76), and dinner (OR 2.13; 95% CI 1.57–2.90). For functional capacity, scores of intellectual activity, social role, competency (total TMIG-IC), social engagement, technology usage, information practice, life management, and total JST-IC were lower in those eating alone across all meals (all *p* < 0.05), with the exception of associations between eating lunch and dinner alone with technology usage and eating alone at lunch with information practice.

## 4. Discussion

This study investigated the associations between eating the three daily meals alone, socio-demographic characteristics, and health status, including functional capacity, in Japanese elderly living with others. The results indicated an association between socio-demographic characteristics and eating alone. In addition, a negative association was observed between eating alone at each meal and various aspects of health status, which differed according to the three daily meals.

Considering socio-demographic characteristics, multiple variables were associated with eating alone. By gender, women were more likely to eat alone at each meal, which is consistent with the findings of previous studies examining the elderly in the United States [22]. In terms of age, those aged 65–69 years were more likely to eat alone at breakfast and lunch than those aged 70–79 years. In Japan, the employment rate of those aged 65–69 years is 54.8% for men and 34.4% for women [34]. The number of respondents eating alone at breakfast and lunch tended to be higher if working, so it is possible that the association with age may be influenced by working status. However, those aged 80 years or over were more likely to eat alone at dinner than those aged 65–69 years. Although cohabitation situations differ, a study of older Japanese people living alone found that those aged 80 years or over were more likely to eat alone [9]. According to the Survey on Time Use and Leisure Activities in Japan, although elderly people aged 75 years and older begin their breakfast approximately 10 min later than the average, this age group begins their dinner approximately 60 min earlier for men and 40 min earlier for women [35]. Therefore, a discrepancy in meal times, relative to cohabitants, affects the proportion of those eating alone even though they are living with others. Those with nine years of education or less were more likely to eat alone at dinner, and those with difficulty in self-assessed living conditions were more likely to eat alone at breakfast. The overall trend was similar to the data reported from previous studies of those living alone [9]. In residential areas, those in new residential areas were more likely to eat alone at lunch, which was similar to the findings of previous studies in the United States [22]. Those in former village areas, however, were more likely to eat alone at breakfast. The difference in employment rate between those living in former village areas versus those living in new residential areas (30% and 20%, respectively; *p* < 0.001) may have affected the probability of eating alone at lunch.

In terms of health status, previous studies showed that the mean TMIG-IC was 11.7 for those 70–74 years old and 10.7 for those 75–79 years old [30], and JST-IC was 10.9 for men and 10.2 for women 65–74 years old, and 8.8 for men and 7.3 for women 75–84 years old [33]. Considering the five-year difference in TMIG-IC (1.0) and the 10-year difference in JST-IC (2.1–2.9), the present study found a difference in the functional capacity of several years between those who did and did not eat alone. JST-IC is a new scale developed two years ago [33]; therefore, the clinical implications of this difference will need to be discussed in future studies. Additionally, eating alone at breakfast was found to be negatively associated with subjective health, history of hypertension, and technology usage in terms of functional capacity. Previous studies in Japan and the UK have shown negative associations of eating alone with subjective health [9] and sodium intake [17], though this may have been particularly influenced by eating alone at breakfast. Although technology usage, a subscale of the JST-IC that asks about the ability to use technology such as mobile phones [33], was found to be associated with eating alone only at breakfast, it can be considered an important functional capacity for any meal when it is difficult to eat together face to face. BMI was not found to be associated with eating alone (even in analyses categorized by underweight and obese). Because depressive symptoms and other functional capacities were negatively associated with eating alone at breakfast, lunch, and dinner, it may be preferable to eat with others at each meal from the viewpoint of frailty prevention, independent of energy intake.

This study has several limitations. First, because this is a cross-sectional study using the baseline survey of the KAGUYA study, the causal relationship between eating alone and health status cannot be examined. For example, for the positive association between eating alone at lunch and history of dyslipidemia, the direction of causality between these variables may be in reversal direction. Second, the frequency of eating alone was not confirmed in this study because a simple self-administered questionnaire was used. However, many aspects of health status, including depressive symptoms, showed results consistent with those of previous studies [10,11,12], suggesting that at least the actual condition of eating alone was captured. Third, the characteristics of the person(s) living with the participants and dining partner(s) were not investigated. In a previous study of elderly Japanese people [36], the frequency with which respondents were eating with various dining partners was reported in the following order: spouse, children, and grandchildren in those living with others, and children, friends, and others in those living alone. Future research should examine the health status in which the association with eating alone is observed according to diet, considering the dining partner. Fourth, it is not known whether the respondents want to eat meals with others. In a previous study of Japanese young adults [37], some respondents indicated that their impression of eating alone was not only lonely but also quick and carefree. In Japan, an increase in the percentage of citizens participating in eating together in their communities is one of the target aims of the current promotion of *shokuiku* (food and nutrition education) [38]. Because those living alone, who were excluded from this study, reported rarely eating with others, this study was unable to examine the association with health status in those living alone. It is, therefore, important to make efforts to increase the region’s percentage of people who want to eat together. Fifth, because medical history and BMI were self-reported, there may have been recall bias. Finally, there is limited generalizability because the setting of this study was limited to a single town. However, because the town encompasses both former village areas and new residential areas, the obtained data indicate that the observed phenomena apply to both urban and rural areas.

## 5. Conclusions

This study investigated the association between eating alone and health status for each meal among community-dwelling Japanese elderly living with others. It was found that depressive symptoms and many subscales of functional capacity were associated with eating alone for each of the three daily meals. Poor subjective health and a history of hypertension were also related to eating alone at breakfast. Future research needs to examine the effects of eating with others at each meal on the health of elderly people, including those living alone.

## Figures and Tables

**Table 1:** Association of socio-demographic characteristics with eating alone at each meal (*n* = 2809).

		Eating Alone at Breakfast ^a^			Eating Alone at Lunch ^a^			Eating Alone at Dinner ^a^		
	*n*	*n*	OR		*n*	OR		*n*	OR	
		%	(95% CI)	*p* ^b^	%	(95% CI)	*p* ^b^	%	(95% CI)	*p* ^b^
Gender										
Men	1378	358	1		330	1		98	1	
		26.0	(ref)		23.9	(ref)		7.1	(ref)	
Women	1431	438	1.26		423	1.33		139	1.41	
		30.6	(1.07–1.48)	**	29.6	(1.13–1.58)	**	9.7	(1.07–1.84)	*
Age										
65–69 year	1062	334	1		328	1		83	1	
		31.5	(ref)		30.9	(ref)		7.8	(ref)	
70–74 year	748	176	0.67		160	0.61		43	0.72	
		23.5	(0.54–0.83)	***	21.4	(0.49–0.76)	***	5.7	(0.49–1.05)	
75–79 year	511	122	0.68		121	0.69		49	1.25	
		23.9	(0.54–0.87)	**	23.7	(0.55–0.88)	**	9.6	(0.86–1.81)	
80 year or older	488	164	1.10		144	0.94		62	1.72	
		33.6	(0.88–1.39)		29.5	(0.74–1.18)		12.7	(1.21–2.43)	**
Education history										
9 year or less	656	199	1		178	1		75	1	
		30.3	(ref)		27.1	(ref)		11.4	(ref)	
10–12 year	1274	352	0.88		346	1.00		100	0.66	
		27.6	(0.71–1.08)		27.2	(0.81–1.24)		7.8	(0.48–0.90)	*
13 year or more	860	243	0.90		227	0.96		61	0.59	
		28.3	(0.72–1.13)		26.4	(0.77–1.21)		7.1	(0.41–0.84)	**
Self-assessed living conditions ^c^										
Difficult	518	166	1		152	1		49	1	
		32.0	(ref)		29.3	(ref)		9.5	(ref)	
Not difficult	2270	624	0.80		597	0.86		185	0.85	
		27.5	(0.65–0.99)	*	26.3	(0.70–1.06)		8.1	(0.61–1.18)	
Area of residence										
Former village areas	1607	480	1		390	1		140	1	
		29.9	(ref)		24.3	(ref)		8.7	(ref)	
New residential areas	1188	314	0.84		361	1.36		97	0.93	
		26.4	(0.71–1.00)	*	30.4	(1.15–1.61)	***	8.2	(0.71–1.22)	
Working status										
Not working	2059	564	1		530	1		175	1	
		27.4	(ref)		25.7	(ref)		8.5	(ref)	
Working	710	221	1.20		213	1.24		56	0.92	
		31.1	(0.99–1.44)		30.0	(1.02–1.49)	*	7.9	(0.67–1.26)	

OR, odds ratio; CI, confidence interval. ^a^ 0 = eating together, 1 = eating alone. ^b^ Binary logistic regression analysis. ^c^ Difficult: "very difficult, difficult", not difficult: "normal, somewhat comfortable, very comfortable". * *p* < 0.05. ** *p* < 0.01. *** *p* < 0.001.

**Table 2:** Association of eating alone at each meal with subjective health, medical history, care needs, and depression (*n* = 2809).

		(Eating Breakfast)			(Eating Lunch)			(Eating Dinner)		
		Together	Alone	*p* ^a^	Together	Alone	*p* ^a^	Together	Alone	*p* ^a^
Subjective health ^bc^	*n*	2004	792		2048	748		2560	236	
Not in good health	*n*	290	139		311	118		384	45	
	%	14.5	17.6		15.2	15.8		15.0	19.1	
	OR	1	1.27		1	1.10		1	1.19	
	(95% CI)	(ref)	(1.01–1.61)	*	(ref)	(0.86–1.41)		(ref)	(0.83–1.73)	
Medical history ^b^	*n*	2004	792		2047	749		2561	235	
Hypertension	*n*	826	370		872	324		1090	106	
	%	41.2	46.7		42.6	43.3		42.6	45.1	
	OR	1	1.26		1	1.06		1	1.02	
	(95% CI)	(ref)	(1.06–1.49)	**	(ref)	(0.89–1.26)		(ref)	(0.77–1.35)	
Diabetes	*n*	269	116		265	120		349	36	
	%	13.4	14.6		12.9	16.0		13.6	15.3	
	OR	1	1.13		1	1.38		1	1.15	
	(95% CI)	(ref)	(0.88–1.43)		(ref)	(1.08–1.75)	*	(ref)	(0.78–1.70)	
Cardiovascular disease	*n*	221	95		232	84		285	31	
	%	11.0	12.0		11.3	11.2		11.1	13.2	
	OR	1	1.16		1	1.08		1	1.25	
	(95% CI)	(ref)	(0.88–1.51)		(ref)	(0.82–1.42)		(ref)	(0.83–1.90)	
Stroke	*n*	52	16		56	12		63	5	
	%	2.6	2.0		2.7	1.6		2.5	2.1	
	OR	1	0.79		1	0.59		1	0.85	
	(95% CI)	(ref)	(0.45–1.41)		(ref)	(0.31–1.15)		(ref)	(0.33–2.17)	
Osteoporosis	*n*	140	72		150	62		185	27	
	%	7.0	9.1		7.3	8.3		7.2	11.5	
	OR	1	1.15		1	1.02		1	1.29	
	(95% CI)	(ref)	(0.84–1.58)		(ref)	(0.74–1.42)		(ref)	(0.82–2.04)	
Rheumatoid arthritis	*n*	57	18		55	20		64	11	
	%	2.8	2.3		2.7	2.7		2.5	4.7	
	OR	1	0.78		1	1.01		1	1.86	
	(95% CI)	(ref)	(0.45–1.34)		(ref)	(0.60–1.71)		(ref)	(0.96–3.61)	
Dyslipidemia	*n*	746	286		779	253		955	77	
	%	37.2	36.1		38.1	33.8		37.3	32.8	
	OR	1	0.93		1	0.80		1	0.85	
	(95% CI)	(ref)	(0.78–1.10)		(ref)	(0.67–0.96)	*	(ref)	(0.64–1.14)	
Care needs ^b^	*n*	2010	795		2052	753		2568	237	
Certification	*n*	102	63		107	58		137	28	
	%	5.1	7.9		5.2	7.7		5.3	11.8	
	OR	1	1.24		1	1.39		1	1.23	
	(95% CI)	(ref)	(0.85–1.82)		(ref)	(0.95–2.05)		(ref)	(0.73–2.10)	
GDS 5 ^bd^	*n*	1909	765		1951	723		2448	226	
Depressive symptoms	*n*	426	244		458	212		578	92	
	%	22.3	31.9		23.5	29.3		23.6	40.7	
	OR	1	1.71		1	1.43		1	2.13	
	(95% CI)	(ref)	(1.40–2.09)	***	(ref)	(1.17–1.76)	**	(ref)	(1.57–2.90)	***

OR, odds ratio; CI, confidence interval; GDS 5, 5-item Geriatric Depression Scale. ^a^ Binary logistic regression analysis adjusted for gender, age, education history, self-assessed living conditions, residential area, and working status. ^b^ 0 = Absence of symptoms, 1 = Presence of symptoms. ^c^ Not in good health: "bad, not good", in good health: "not bad, good, very good. ^d^ Two or more negative answers for depression screening were used to diagnose the presence of depressive symptoms. * *p* < 0.05. ** *p* < 0.01. *** *p* < 0.001.

**Table 3:** Association of eating alone at each meal with BMI and scores of functional capacity (*n* = 2809).

		(Eating Breakfast)			(Eating Lunch)			(Eating Dinner)		
		Together	Alone	*p* ^a^	Together	Alone	*p* ^a^	Together	Alone	*p* ^a^
BMI: weight (kg)/height (m)^2^	*n*	1913	767		1951	729		2455	225	
	Mean	22.7	22.7	0.890	22.7	22.7	0.627	22.7	22.4	0.570
	SD	3.0	3.0		2.9	3.0		3.0	3.0	
Functional capacity										
TMIG-IC										
Instrumental Self-Maintenance ^b^	*n*	1916	765		1954	727		2457	224	
	Mean	4.5	4.5	0.818	4.5	4.6	0.554	4.5	4.3	0.230
	SD	1.1	1.2		1.1	1.1		1.1	1.4	
Intellectual Activity ^c^	*n*	1917	759		1957	719		2454	222	
	Mean	3.5	3.4	0.001	3.5	3.4	0.040	3.5	3.2	< 0.001
	SD	0.8	0.9		0.8	0.9		0.8	1.1	
Social Role ^c^	*n*	1892	761		1937	716		2435	218	
	Mean	3.0	2.8	< 0.001	3.0	2.8	< 0.001	3.0	2.6	< 0.001
	SD	1.1	1.2		1.1	1.2		1.1	1.3	
TMIG-IC score ^d^	*n*	1847	736		1889	694		2375	208	
	Mean	11.1	10.7	< 0.001	11.0	10.9	0.004	11.1	10.2	< 0.001
	SD	2.4	2.6		2.4	2.5		2.4	3.2	
JST-IC										
Social Engagement ^c^	*n*	1896	753		1931	718		2429	220	
	Mean	1.9	1.5	< 0.001	1.8	1.5	< 0.001	1.8	1.2	< 0.001
	SD	1.6	1.5		1.6	1.5		1.6	1.5	
Technology Usage ^c^	*n*	1884	750		1921	713		2415	219	
	Mean	2.9	2.8	0.039	2.9	2.9	0.942	2.9	2.6	0.486
	SD	1.3	1.4		1.4	1.4		1.4	1.5	
Information Practice ^c^	*n*	1874	747		1916	705		2403	218	
	Mean	3.1	2.9	0.003	3.0	3.0	0.078	3.0	2.7	0.002
	SD	1.1	1.2		1.2	1.2		1.1	1.3	
Life Management ^c^	*n*	1848	738		1885	701		2374	212	
	Mean	2.9	2.6	< 0.001	2.9	2.7	< 0.001	2.9	2.4	< 0.001
	SD	1.2	1.3		1.2	1.3		1.2	1.4	
JST-IC score ^e^	*n*	1746	692		1772	666		2238	200	
	Mean	10.3	9.4	< 0.001	10.1	9.7	< 0.001	10.1	8.6	< 0.001
	SD	3.6	3.7		3.7	3.6		3.6	4.0	

BMI, body mass index; SD, standard deviation; TMIG-IC, Tokyo Metropolitan Institute of Gerontology Index of Competence; JST-IC, Japan Science and Technology Agency Index of Competence. ^a^ Analysis of covariance adjusted for gender, age, education history, self-assessed living conditions, residential area, and working status. ^b^ Range: 0 (Not good)–5 (Good). ^c^ Range: 0 (Not good)–4 (Good). ^d^ Range: 0 (Not good)–13 (Good). ^e^ Range: 0 (Not good)–16 (Good).
